# Measuring Physical Activity with Hip Accelerometry among U.S. Older Adults: How Many Days Are Enough?

**DOI:** 10.1371/journal.pone.0170082

**Published:** 2017-01-12

**Authors:** Masha Kocherginsky, Megan Huisingh-Scheetz, William Dale, Diane S. Lauderdale, Linda Waite

**Affiliations:** 1 University of Chicago Medicine, Department of Public Health Sciences, Chicago, IL, United States of America; 2 University of Chicago Medicine, Section of Geriatrics and Palliative Medicine, Chicago, IL, United States of America; 3 University of Chicago Medicine, Department of Sociology, Chicago, IL, United States of America; Universite de Nantes, FRANCE

## Abstract

**Introduction:**

Accelerometers are increasingly used in research. Four to 7 days of monitoring is preferred to estimate average activity but may be burdensome for older adults. We aimed to investigate: 1) 7-day accelerometry protocol adherence, 2) demographic predictors of adherence, 3) day of the week effect, and 4) average activity calculated from 7 versus fewer days among older adults.

**Methods:**

We used the 2003–2006 older adult hip accelerometry data from the National Health and Nutrition Examination Survey (NHANES) sample. We determined proportions with 1–7 valid (10–20 hours) wear days and identified wear day correlates using ordinal logistic regression. We determined the day of week effect on 5 accelerometry measures (counts per minute, CPM; % sedentary behavior; % light-lifestyle activity; % moderate-vigorous activity, MVPA; total activity counts) using multivariate linear regression and compared averages estimated over 2 or 3 versus 7 days using correlations, linear regression, and Bland-Altman plots.

**Results:**

Among 2,208 participants aged 65+, 85% of participants had ≥2 and 44% had 7 valid wear days. Increasing age (*p* = 0.01) and non-white race (*p* < 0.001) were associated with fewer days. Daily CPM, % MVPA, and total daily activity counts were similar Monday through Saturday, but significantly lower on Sundays *(p* < 0.001). Daily % sedentary behavior and % light-lifestyle activity were significantly different on Saturdays (p = 0.04–0.045) and Sundays (p < 0.001) compared to weekdays. Among participants with 7 valid days, 2 or 3 day averages were highly correlated with 7 day averages for all 5 accelerometry measures (2 versus 7 days: *r* = 0.90–0.93, 3 versus 7 days: *r* = 0.94–0.96).

**Conclusions:**

Protocols of 2–3 days, adjusting for Sundays (average CPM, % moderate-vigorous activity, and average total daily activity counts) or weekends (% sedentary behavior and % light-lifestyle activity), give reliable estimates of older adult activity.

## Introduction

Survey questions about physical activity are difficult for older respondents to answer accurately [1–3]. Monitors that estimate activity level using accelerometers are increasingly used in research to provide an objective estimate of activity. They may be helpful to characterize overall physical activity level, monitor intervention program adherence, and/or identify fall risk or frailty behaviors.[4, 5]

Current expert recommendations suggest that ~4–7 consecutive days (and as few as 3.5 days) of accelerometer measurement are necessary to accurately assess physical activity patterns in adults.[6, 7] For older adults, others suggest that requiring 7 days of monitoring may be overly burdensome, may decrease adherence for clinical applications, or introduce a selection bias in research applications.[8, 9] A major concern with using fewer than 7 days is the introduction of bias due to potential systematic differences in activity by day of the week, especially on weekends. However, day-of-the-week effects may be different for older adults. Older adults are more likely to be retired, which may decrease their weekday-weekend activity variability. Limited mobility due to health or impairments may also result in more consistent day-to-day activity patterns.[10–12] These unique features among older adults suggest that fewer than 7 days of accelerometry monitoring may adequately reflect typical activity patterns for this population.

To explore these issues, we used the 2003–4 and 2005–6 National Health and Nutrition Examination Survey (NHANES), collected by the National Center for Health Statistics, and designed to be nationally representative of the community-dwelling population. Those two biennial cycles of NHANES included a seven-day protocol using hip accelerometry. We focus on adults aged 65 and older. We hypothesized that in a nationally-representative sample of older adults, 7-day protocol adherence would be poor, that older ages would be associated with fewer valid days, that activity levels would be similar over each day of the week, and that activity measures collected over 2 or 3 valid days of wear would closely correlate with those collected over 7 valid days.

Our analyses in a nationally-representative sample of older adults addresses 4 key unresolved issues about measuring activity levels with accelerometry. First, regarding whether concerns about 7-day protocol adherence among older adults are justified, we determine the 7-day protocol adherence among only the older adults. Second, regarding whether age or other demographic variables predict adherence [9], we characterize the sampling biases which could be introduced by non-adherence to longer protocols. Third, regarding whether shortened accelerometry protocols would introduce weekend/weekday activity variation bias, we assess whether and how activity patterns vary by day of the week. Finally, regarding whether less than 7 days of wear time is adequate to estimate activity levels, we compare 7 days with fewer than 7 days wear time.

## Materials and Methods

### National Health and Nutrition Examination Survey (NHANES)

NHANES is a nationally representative survey of the civilian, non-institutionalized, U.S. population, and consists of questionnaires administered in the home, followed by a physical examination in a specially equipped mobile examination center. NHANES uses a complex, multistage probability sampling design [13] oversampling certain subgroups, including Mexican-American and Black persons and older adults (age ≥ 70). NHANES interviews and examines a nationally-representative sample of approximately 5,000 persons each year; data are released in 2-year cycles, and combining 2-year cycles also yields nationally representative samples (http://www.cdc.gov/nchs/tutorials/nhanes/SurveyDesign/Weighting/Task2.htm).

### Covariates

Standard demographics, including age (calculated from the birth date or imputed), gender, race/ethnicity, working status and examination period (a 6-month time period indicator, either November 1 through April 30, or May 1 through October 31), were collected for each respondent.

### Accelerometery Measures

Physical activity data were collected in a sub-study in the 2003–2004 and 2005–2006 cycles using a uniaxial physical activity monitor, ActiGraph AM-7164 (formerly the CSA/MTI AM-7164), manufactured by ActiGraph of Ft. Walton Beach, FL. Participants over the age of 6 were randomly assigned to this physical activity monitor sub-study, and subjects who used wheelchairs or had other impairments that prevented them from walking or wearing the accelerometer were excluded. Consenting participants received a physical activity monitor and were asked to wear the device for 7 consecutive days. The device was placed on an elasticized fabric belt and worn on the right hip. Subjects were told to keep the device dry (i.e. remove it before swimming or bathing) and to remove the device at bedtime. The monitors were programmed to begin recording activity information for successive 1 minute intervals (epochs) beginning at 12:01 a.m. the day after the health examination. Further details about the study methodology have been previously published, and the full data description is also available [13, 14]. In this study, we selected respondents aged ≥ 65 years.

ActiGraph data are provided as *counts* that are a result of aggregating post-filtered raw accelerometer data over 1-minute epochs. Data were processed and physical activity summary variables were generated from the minute-to-minute accelerometer data using the R package nhanesaccel. Non-wear time was defined as any interval 60 minutes or longer in which all count values were 0. Days of monitoring with 600–1200 minutes (10–20 hours) of wear time were considered valid days for analysis.

For this analysis, we calculated 5 activity measures using the accelerometry output. *(1) Average count per minute (CPM)*, an indicator of general physical activity level, was the primary physical activity measure used. Average CPM was calculated as the total activity count divided by the number of valid minutes, and was calculated for each hour of the day (60 minute intervals), for each valid day, and for the total wear time for each participant. Non-wear time was defined as any interval of 60 minutes or longer in which all counts were 0 and was excluded for all accelerometry measure calculations except the hourly CPM calculation [15]. Hourly CPM for each participant was calculated using all 60 minutes of each clock hour, regardless of wear time, to be able to plot the average 24-hour activity pattern for the sample. We also performed a sensitivity analysis excluding hours with average CPM = 0, which includes hours when the device was removed, such as sleep at night, naps during the day, and bathing if participants followed instructions. The proportion of older adults with 0 hourly CPM was ≥85% during the night (between 12am and 6am), and < 10% during the day between 9am and 7pm, indicating that 0 hourly CPM represented mostly overnight sleep. We also calculated *average percent of day spent in (2) sedentary (<100 counts/min)*, *(3) light-lifestyle (100–2019 counts/min)*, *or (4) moderate-vigorous (≥ 2020 counts/min) activity* for each valid day by summing the minutes spent in each activity intensity divided by the total valid minutes for each valid day and the total wear time. Finally, we calculated *(5) total daily activity counts* for each valid day and *average daily activity counts* over the total wear time. Average daily steps were only available in the 2005–2006 data cycle and were not included in our primary analyses due to the reduced sample size.

### Statistical Analysis

NHANES uses a complex, multi-stage, probability sampling design, which needs to be taken into account in the statistical analysis. For each data cycle, sample weights were adjusted to account for subjects without usable accelerometer data. Sampling weights were set to 0 for subjects with no valid days. For the combined analysis of both 2003–04 and 2005–06 data cycles, 4-year weights were created to account for the two different reference populations. Estimates adjusted for the sample design are representative of the non-institutionalized U.S. population at the midpoint of the combined survey period (i.e. January 1, 2005).

Descriptive statistics included demographic variables, the number of valid wear days and wear time, and accelerometry measures. The distributions of average CPM, percent of time spent in moderate-vigorous activity, and the total daily counts were skewed, so these variables were square-root (CPM) and log-transformed (%MVPA and total daily counts), to satisfy the normality assumption for analyses.

For our first aim, we examined protocol adherence by reporting the number of days the accelerometer was worn for between 10 to 20 hours, which were considered “valid” days. For our second aim, we then used ordinal logistic regression models to identify demographic and activity predictors of the number of valid days of wear.

For our third aim, we explored whether each of the five accelerometry measures (average CPM, percent time spent in sedentary, light-lifestyle, moderate-vigorous activity, and average total daily activity counts) varied by day of the week. We used multivariable linear regression models to examine how these measures varied by day of the week, adjusting for age, gender, race, employment status, examination period, and total wear time. Wald tests were used to test the composite hypotheses that all coefficients associated with a particular categorical predictor are 0. We also plotted hourly CPM estimates by hour of the day and by day of the week to clarify the day-of-the-week effects.

For the fourth aim, we compared the 5 accelerometry measures calculated from the first 2 and the first 3 days of wear to measures calculated from all 7 days among the older adult users who had 7 valid days of wear (n = 966). We examined the within-participant correlation and agreement between the 2- or 3-day and the 7-day average CPM using Pearson’s correlation, Lin’s concordance correlation coefficient[16, 17] and Bland-Altman plots (average CPM only).[18] Correlation coefficients were calculated without accounting for the sample design, and represent correlation within the study sample rather than a national population estimate. As a sensitivity analysis, we also recalculated the 2 day versus 7 day Peason’s and Lin’s concordance correlation varying which 2 days were used to calculate the estimate (Mon/Tue, Fri/Sat, Sat/Sun, or Sun/Mon). We also calculated the Spearman-Brown prophecy as suggested in Trost et al,[6] which provides an estimate of the required number of measurement days based on the intra-class correlation (ICC), setting the target ICC to 0.80. Multivariate linear regression models relating 7-day versus 2- or 3-dayestimates of average CPM were fitted while controlling for wear time [19], and deviance-based pseudo-R^2^ estimate from these models was used as a population estimate of the correlation. Since CPM was square-root transformed and both moderate-vigorous activity and total daily activity counts were log transformed to satisfy the normality assumption for all other analyses, to maintain consistency, correlation coefficients and Bland-Altman plot estimates are also reported on the square-root scale and log scale, respectively. Bland-Altman plots depict the mean of two measures against their difference and can help detect whether the difference is systematically biased or dependent on the activity level.

All statistical analyses were carried out using R.[20] Data were obtained using the nhanesdata package,[21] and processed using the nhanesaccel package.[22] Unless indicated otherwise, all statistical analyses accounted for the complex survey design of NHANES, and survey weights were adjusted for using two waves of data. Analyses were done using the survey package in R.[23, 24]

## Results

### Descriptive Statistics

A total of 2,208 participants aged 65 and older were included in the accelerometer sub-study (Table 1): 1,196 older adult participants in 2003–04 and 1,012 in 2005–06. The weighted proportions were 56.8% (95% CI: 54.8%-58.8%) female; 83.4% (95% CI: 79.5%-87.3%) white; and 84.5% (95% CI: 82.2%-86.9%) not working.

**Table 1:** Sample Characteristics Among Accelerometry Sub-Study Participants aged ≥ 65 (n = 2208) in NHANES 2003 to 2006.

	Estimate	95% Confidence Interval
**Demographics**		
**Gender**		
Women	56.8%	54.8%–58.8%
**Age** (mean, years)	74.2	73.7–74.7
**Race**		
Black	8.1%	5.71%–10.5%
White	83.4%	79.5%–87.3%
Mexican-American	3.24%	1.43%–5.06%
Other	3.2%	2.1%–4.2%
**Employment Status**		
Not Working	84.5%	82.2%–86.9%

Among the 14,435 wear days assessed, 78.4% were valid wear days. (Table 2). Among all participants ≥ 65, 1,979 (89.6%) had at least 1 valid wear day. The average number of valid wear days per participant was 5.8 (95% CI: 5.7–6.0). The average total wear time was 4,933.6 minutes (~82.2 hours) while the average daily wear time was 838.8 minutes (~14.0 hours). Among older adults with 1 or more valid wear days, the average count per minute was 185.1 (95% CI: 177.3–192.9), the average daily total counts was 944,470 counts per day (95% CI: 892,240–996,700), and, the majority of the average day was spent sedentary (67.1%, 95% CI: 66.4–67.8%).

**Table 2:** Sample Hip Accelerometry Measures Among Accelerometry Sub-Study Participants aged ≥ 65 (n = 2208) in NHANES 2003 to 2006.

	Estimate	95% Confidence Interval	5^th^ Percentile	95^th^ Percentile
**Hip Accelerometry Measures**				
Among All Wear Days (n = 14,435):				
Valid Days (N, %)	11,323 (78.4%)	-	-	-
Invalid Days (N,%)	3,112 (21.6%)	-	-	-
Average Number of Valid Wear Days^1^ (mean, SD)	5.8 (1.7)	5.7–6.0	2.0	7.0
Total Valid Minutes (mean, SD)	4,933.6 (1,592.0)	4819.6–5047.5	1347.6	6747.3
Average Daily Valid Minutes (mean, SD)	839.8 (91.8)	832.9–846.7	685.6	985.5
Average Count Per Minute (mean, SD)	185.1 (107.5)	177.3–192.9	52.3	376.1
Average Daily Total Counts (mean, SD)	944,470 (648,600)	892,240–996,700	125,760	2,150,840
Average Daily Sedentary Behavior (%, SD)	67.1 (12.0)	66.4–67.8	46.0	86.9
Average Daily Light-Lifestyle Activity (%, SD)	32.0 (11.5)	31.3–32.6	12–9	51–7
Average Daily Moderate-Vigorous Activity (%, SD)	1.0 (1.5)	0.9–1.1	0.04	4.03

^1^ Among Participants with ≥1 Valid Day (n = 1,979).

### Adherence to Accelerometry Protocol and Predictors of Adherence

**Fig 1:**
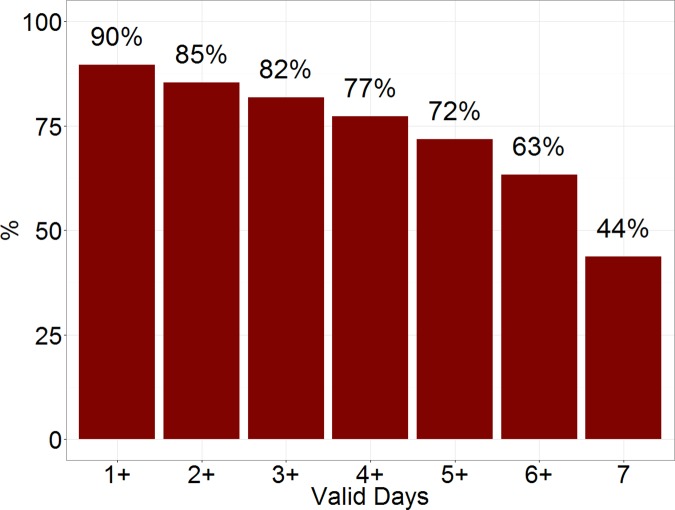
Adherence to the hip accelerometry protocol among a nationally representative sample of older (≥65) adults in the National Health and Nutrition Survey (NHANES).

Among older participants, only 44% had 7 valid wear days, whereas 85% had 2 or more valid days, and 82% had three or more valid days (Fig 1).

Multivariable ordinal logistic regression identified demographic variables associated with the number of valid wear days (S1 Table). The number of valid days decreased with age (OR = 0.89, p = 0.01 per 5 year increase in age) and was significantly lower among non-white older adults (p<0.001; Wald test). Gender, working status, and examination period were not associated with the number of valid days. Age and CPM were negatively correlated (*r* = -0.38), and therefore we were unable to further adjust for CPM in this model. Instead, we re-fitted the model among participants with lower CPM ≤150 (n = 903) and CPM >150 (n = 1,076), and found that age was significantly negatively related to the number of valid days among those with lower activity levels (p = 0.02), but not among those with higher CPM (p = 0.78).

### Activity Patterns by Day of Week and Hour of Day

**Fig 2:**
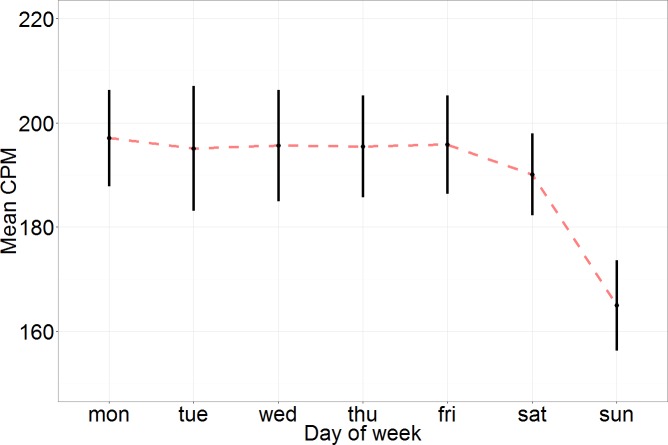
Average daily count per minute (CPM) and 95% confidence intervals by day of the week among adults aged 65 and older with 7 valid days of accelerometry wear in National Health and Nutrition Examination Survey 2003–4 and 2005–6.

Fig 2 shows average daily CPM and 95% confidence intervals by day of the week. On weekdays (Monday through Friday), average CPM varied little across days, ranging from 195.1 to 197.1 but was slightly lower on Saturdays (average CPM: 190.1) and much lower on Sundays (average CPM = 165.0). We found a similar pattern among the average daily percent of time spent in sedentary, light-lifestyle, and moderate-vigorous activity as well as the average total daily activity counts. Monday through Friday sedentary behavior ranged from 65.3–65.9%, while Saturday was slightly higher at 66.3%, and Sunday was much higher at 69.6%. Monday through Friday light-lifestyle activity ranged from 33.1–33.6%, while Saturday was slightly lower at 32.6%, and Sunday was much lower at 29.6%. Monday through Friday moderate-vigorous activity ranged from 1.0–1.1%, while Saturday was similar at 1.0%, and Sunday was slightly less at 0.8%.

Multivariable linear regression models examined the day of the week effect on each of the 5 accelerometry measures while adjusting for age, gender, race, employment status, examination period, and wear time. Average CPM was significantly different on Sundays compared to Monday through Saturday (p<0.001) and not significantly different on Saturday compared to Monday through Friday (p = 0.08). The reduced activity on Sundays was evident for both genders (S1A Fig), and persisted regardless of employment status (S1B Fig). Percent daily sedentary behavior was significantly different on Sundays compared to Monday through Saturday (p<0.001) and on Saturdays compared to Monday through Friday (p = 0.045) as well as between Saturdays compared to Sundays (p<0.001). Light-lifestyle was significantly different on Sundays compared to Monday through Saturday (p<0.001) and on Saturdays compared to Monday through Friday (p = 0.04) as well as between Saturdays compared to Sundays (p<0.001). Moderate-vigorous activity was significantly different on Sundays compared to Monday through Saturday (p<0.001) but not significantly different on Saturdays compared to Monday through Friday (p = 0.11). Average total daily activity counts was significantly different on Sundays compared to Monday through Saturday (p<0.001) but not significantly different on Saturdays compared to Monday through Friday (p = 0.08).

**Fig 3:**
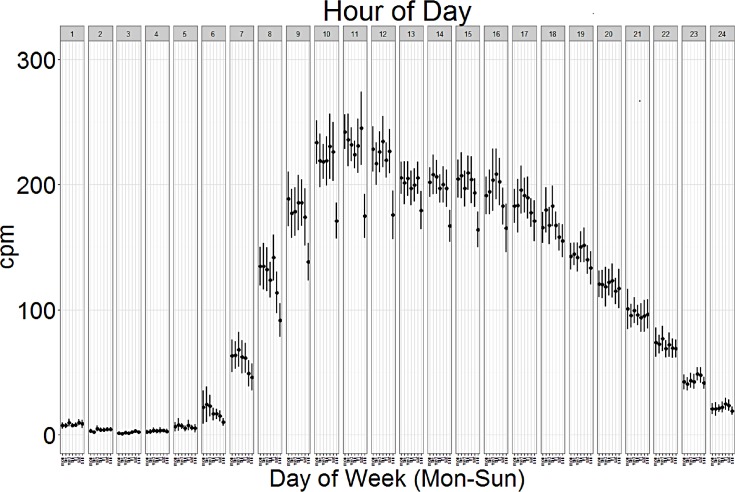
Hourly activity (counts per minute, CPM) by day of week among adults aged 65 and older in National Health and Nutrition Examination Survey 2003–4 and 2005–6 accelerometry sub-study.

Mean hourly CPM by day of the week are shown in Fig 3 to illustrate the day of week effect in more detail. Each panel represents average CPM during the corresponding hour of the day beginning at midnight (e.g. hour 1 represents activity between midnight and 1:00 am) throughout the week. Overall, older adults were most active between the hours of 9:00 am and 12:00 pm. For any given hour of the day, activity levels were similar Monday through Saturday, and were much lower on Sundays most notably between 9:00 am and 12:00 pm.

### Comparing Two or Three Valid Wear Days Versus Seven Valid Wear Days

**Fig 4:**
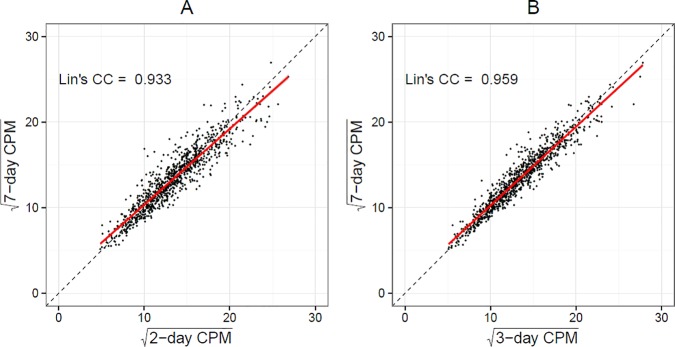
Correlation and Pearson’s Correlation Coefficient between 2-day (A) and 3-day (B) average counts per minute versus 7-day average counts per minute among adults aged 65 and older in National Health and Nutrition Examination Survey 2003–4 and 2005–6 accelerometry sub-study.

**Fig 5:**
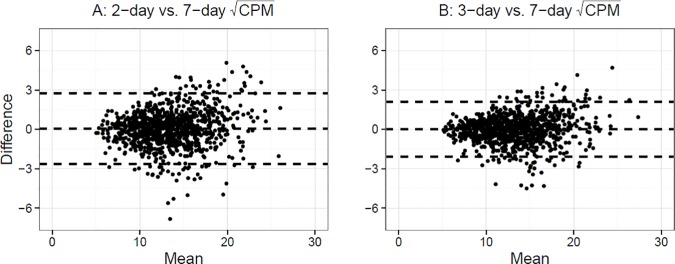
Bland-Altman agreement plots between 2-day (A) and 3-day (B) $\sqrt{counts\;per\;minutes}$ versus 7-day $\sqrt{counts\;per\;minute}$ among adults aged 65 and older in National Health and Nutrition Examination Survey 2003–4 and 2005–6 accelerometry sub-study.

Average within-participant difference between the 7-day average CPM and 2- or 3-day average CPM was -2.9 (95% CI: -5.8–0.028) and -1.8 (95% CI: -4.0–0.44), respectively, and were not significantly different from 0 (p = 0.2 and p = 0.5). Lin's concordance correlation coefficient [16] was 0.93 for 2-day vs. 7-day average CPM, and 0.96 for 3-day vs. 7-day average CPM (Fig 4). Bland-Altman plots similarly indicated excellent agreement between the measures (Fig 5). Although the differences between measures are larger at higher activity levels, the mean difference remained small and close to 0 for the entire range of CPM, indicating lack of systematic bias. Survey-weighted linear regression models controlling for wear time and predicting 7-day average CPM from 2- or 3-day average CPM produced slope estimates close to 1 (β_2d_ = 0.89 (95% CI: 0.87–0.91) and β_3d_ = 0.92 (95% CI: 0.9–0.94)), and the deviance-based pseudo-R^2^ was 0.87 and 0.92 for the two models, again suggesting high correlation between the 2 or 3 day and 7 day average CPM measures. Among the n = 966 subjects with 7 days of observation, the estimated ICC was 0.68 indicating low within person activity variability across days, and the Spearman-Brown prophecy for the target ICC = 0.80 would require k = 1.88 days of monitoring, which is at least 2 days. Excluding Sundays, the Spearman-Brown prophecy was similar (k = 1.82).

We similarly compared the 2- or 3- day averages to 7-day averages for percent of time spent in sedentary, light-lifestyle, and moderate-vigorous activity, as well as for total daily activity counts. We found similarly high Lin’s concordance coefficients between the pairs (S2 Fig). Lin’s correlation coefficients were high: 0.91 and 0.94 for sedentary activity, 0.91 and 0.94 for life-lifestyle activity, 0.90 and 0.94 for moderate-vigorous activity, and 0.92 and 0.95 for total daily activity counts for the 2 versus 7 and 3 versus 7 day comparisons, respectively.

As a sensitivity analysis, we determined whether the Lin's concordance correlation coefficients were affected by which pair of days was considered. Among participants with the full 7 days, we compared the correlation between Monday/Tuesday, Friday/Saturday, Saturday/Sunday, and Sunday/Monday estimates versus the 7day estimates. We found that the Lin’s concordance correlations coefficients are still reasonably high, but do differ depending on which two days are selected, with the Saturday/Sunday estimates versus the 7 day estimates having the lowest correlation. Across the 2-day combinations, correlation coefficient ranged as follows: average CPM Lin’s = 0.90–0.94; % sedentary activity Lin’s *r* = 0.89–0.92; % light-lifestyle activity Lin’s *r* = 0.89–0.92; % moderate-vigorous activity Lin’s *r* = 0.87–0.91; total daily activity counts Lin’s *r* = 0.89–0.94.

## Discussion

We used a nationally-representative sample of older adults from the NHANES 2003–04 and 2005–06 hip accelerometry study to determine whether daytime accelerometry protocols with fewer than the recommended 7 days were sufficient for physical activity assessment. We confirmed in a national sample that adherence to a 7-day accelerometery protocol among older adults was low, with only 43.8% providing the full 7 days of accelerometry data including at least 10 hours of daily wear time. However, adherence was much higher for 2 days, with 85.3% having two or more valid wear days. Older adults largely wore the accelerometer for the entire day (at least 10 hours) when they wore the device at all. Therefore, non-adherence among older adults is primarily driven by the requirement to wear the device for many days, rather than by having to wear it for long periods of time. Furthermore, both increasing age and minority race status were associated with fewer valid wear days, suggesting that restricting accelerometry analyses to participants with 7 valid days would under-represent these important sub-groups.

As with children and younger adults,[25] older adults are less active on weekends, especially on Sundays. For older adults, accelerometry measures of sedentary behavior (%) and light-lifestyle activity (%) are significantly different on both Saturdays and Sundays compared to weekdays. However, unlike children and younger adults, the activity reduction was only statistically significant on Sundays compared to the rest of the week for average CPM, moderate-vigorous activity (%), and total daily activity counts. Average CPM had a distinct pattern of being lowest on Sunday mornings and early afternoons. These findings have implications for the accuracy of future accelerometry research protocols.

To determine whether a shorter accelerometry protocol is sufficient to accurately estimate daily activity, we focused on the subset of participants with 7 valid days of accelerometry data, and re-estimated their activity levels using only their first 2 and 3 days. All 5 accelerometry measures based on the first 2 or 3 days of observation were very highly correlated (>0.90) with 7 days, suggesting that fewer days show excellent overall agreement with the full evaluation summaries. Our findings suggest that, for older adults, it is reasonable for accelerometry protocols to include as few as 2 or 3 wear days when estimating average activity levels. Shorter protocols will improve adherence and reduce selection bias while maintaining reasonable validity. In contrast, if the goal is to estimate activity levels for an entire week, or to compare activity levels with other, longer studies or those with established cut points, using a 2- or 3-day protocol excluding Sundays for average CPM, % moderate-vigorous activity, and total daily activity count analyses, or excluding weekends for % sedentary behavior and % moderate-vigorous activity analyses, will very slightly overestimate the subject's average weekly activity levels when compared to the full 7-day estimate, or a shorter one that includes the weekends.

These findings should be considered in light of the study’s limitations. First, our analysis explored activity among the older adult sub-sample, so our results do not apply to adults under the age of 65. We also compared 2- and 3-day accelerometry measures among those older adults who were 7-days *adherent*. In our analyses, we found that this subset of older adults was younger and included fewer minorities than the non-adherent subset. Therefore, the relationship between the 2- or 3-day estimates and the full 7-day estimates may not be the same among non-compliers lacking all 7 days of wear.

Studying activity patterns among older, frail, multimorbid adults is important, but adherence to a 7-day accelerometry protocol may be especially challenging for this population. To increase study participation and include harder-to-reach populations, we recommend relaxing wear time requirements to 2 or 3 days of accelerometry when studying average activity levels. We also recommend consistently including or excluding Sunday and Saturday, depending on the goals of the study and the accelerometry measure being used. We believe the cost savings and increased protocol adherence will outweigh the gain in precision for longer protocols in most clinical and research applications among older adults.

## Supporting Information

S1 Table Multivariable Ordinal Logistic Regression Model of Number of Valid Accelerometer Wear Days in the Accelerometry Sub-Study Participants aged ≥ 65 (n = 2208) in NHANES 2003 to 2006. (DOCX)

S1 Fig Average daily counts per minute and 95% confidence intervals by gender (A) and employment status (B) among adults aged 65 and older in National Health and Nutrition Examination Survey 2003–4 and 2005–6 accelerometry sub-study.
(TIFF)

S2 Fig Correlation and Lin’s concordance correlation coefficients between 2-day (A) and 3-day (B) average percent of time spent in sedentary, light-lifestyle, and moderate-vigorous activity per daily versus 7-day estimate among adults aged 65 and older in National Health and Nutrition Examination Survey 2003–4 and 2005–6 accelerometry sub-study.
(TIFF)
