# Correction: Measuring Physical Activity with Hip Accelerometry among U.S. Older Adults: How Many Days Are Enough?

**DOI:** 10.1371/journal.pone.0174739

**Published:** 2017-03-22

**Authors:** Masha Kocherginsky, Megan Huisingh-Scheetz, William Dale, Diane S. Lauderdale, Linda Waite

There is an error in the second sentence of the fourth paragraph of the Materials and Methods section under the subheading “Statistical Analysis.” The correct sentence is: We examined the within-participant correlation and agreement between the 2- or 3-day and the 7-day average CPM using Lin’s concordance correlation coefficient [16, 17] and Bland-Altman plots (average CPM only).

There are errors in the caption for Fig 4. Please see the complete, correct Fig 4 caption here.

**Fig 4:**
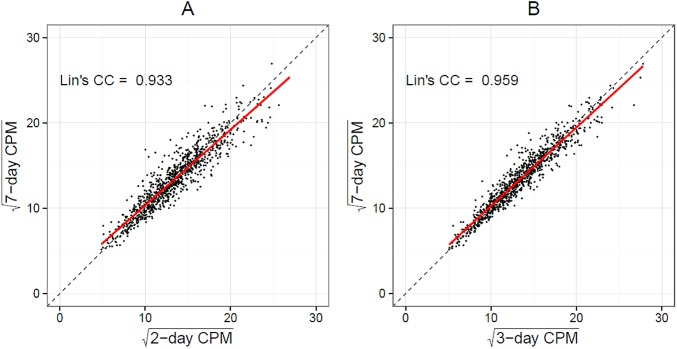
Lin’s concordance correlation coefficients between 2-day (A) and 3-day (B) average counts per minute versus 7-day average counts per minute among adults aged 65 and older in National Health and Nutrition Examination Survey 2003±4 and 2005±6 accelerometry sub-study.

There are errors in the caption for S2 Fig. Please see the complete, correct S2 Fig caption here.

## Supporting information

S2 Fig Lin's concordance correlation coefficients between 2-day (A) and 3-day (B) average percent of time spent in sedentary, light-lifestyle, and moderate-vigorous activity per daily versus 7-day estimate among adults aged 65 and older in National Health and Nutrition Examination Survey 2003±4 and 2005±6 accelerometry sub-study.
(TIFF)
