# Supplementary material for: Measuring Physical Activity with Hip Accelerometry among U.S. Older Adults: How Many Days Are Enough?
Source: PLoS One. 2017 Jan 12;12(1):e0170082. doi: 10.1371/journal.pone.0170082 (PMC5231361; doi:10.1371/journal.pone.0170082)
Supplement: S1 Table — (DOCX) [file pone.0170082.s001.docx]

|  | OR | p-value |
| --- | --- | --- |
| **Age (per 5-year interval)** | 0.89 | 0.01 |
| **Gender** |  |  |
| Women | 0.94 | 0.54 |
| **Race** |  | 0.001 |
| Black | 0.49 |  |
| Mexican-American | 0.71 |  |
| White | Ref. |  |
| **Employment Status** |  |  |
| Working | 0.92 | 0.58 |
