# Supplementary figures and images for: Measuring Physical Activity with Hip Accelerometry among U.S. Older Adults: How Many Days Are Enough?
Source: PLoS One. 2017 Jan 12;12(1):e0170082. doi: 10.1371/journal.pone.0170082 (PMC5231361; doi:10.1371/journal.pone.0170082)

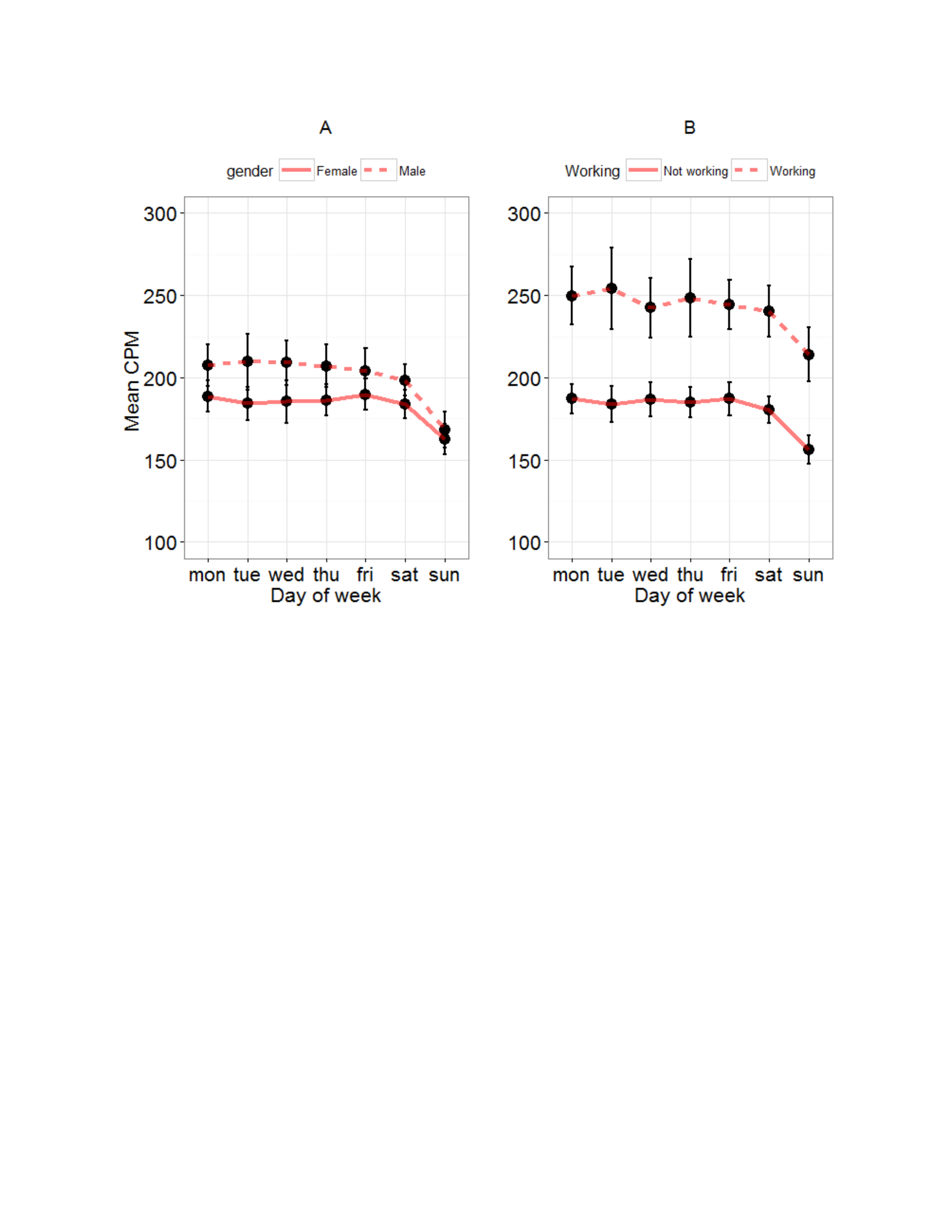

Supplement: S1 Fig — Average daily counts per minute and 95% confidence intervals by gender (A) and employment status (B) among adults aged 65 and older in National Health and Nutrition Examination Survey 2003–4 and 2005–6 accelerometry sub-study. (TIFF) [file pone.0170082.s002.tiff]

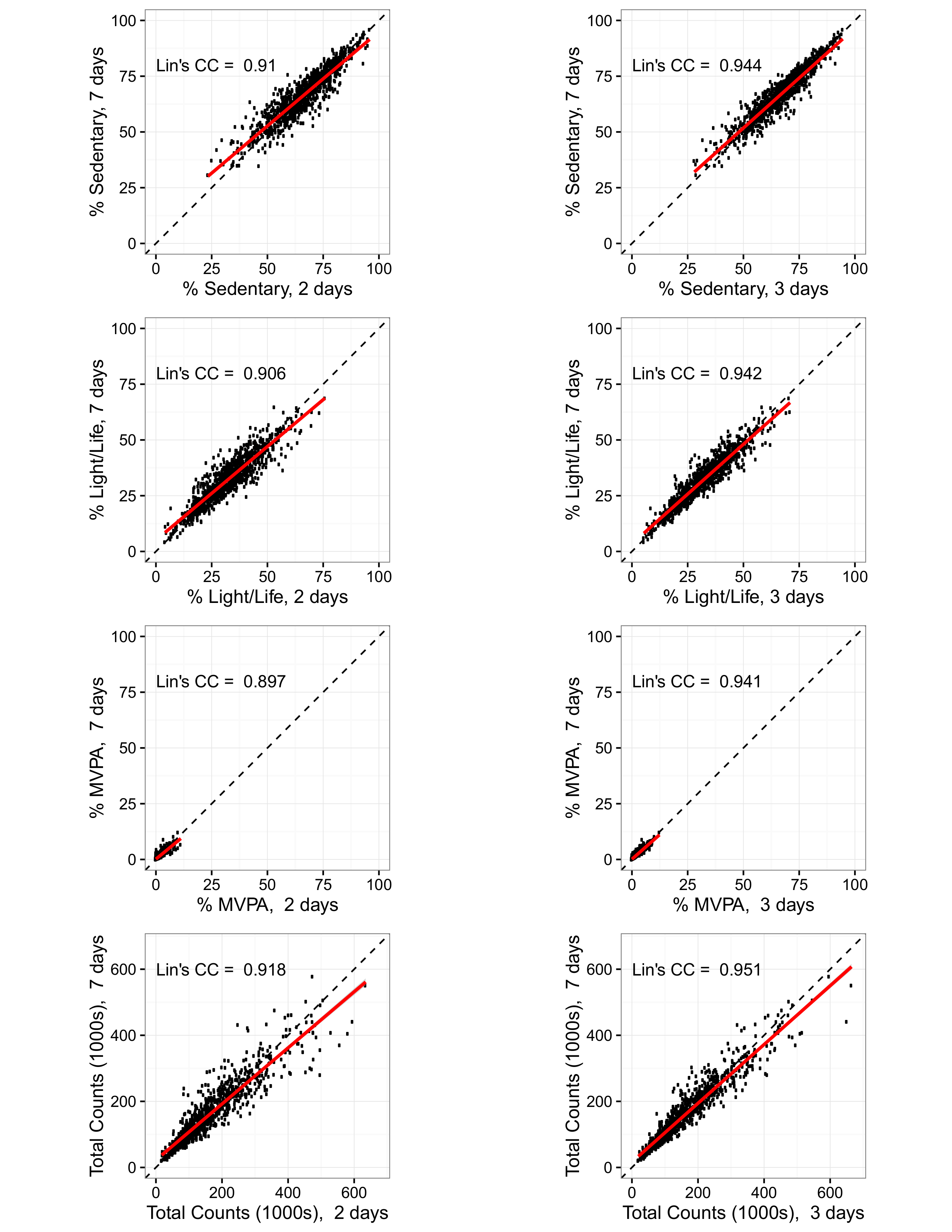

Supplement: S2 Fig — Correlation and Lin’s concordance correlation coefficients between 2-day (A) and 3-day (B) average percent of time spent in sedentary, light-lifestyle, and moderate-vigorous activity per daily versus 7-day estimate among adults aged 65 and older in National Health and Nutrition Examination Survey 2003–4 and 2005–6 accelerometry sub-study. (TIFF) [file pone.0170082.s003.tiff]
