# Supplementary figures and images for: Correction: Measuring Physical Activity with Hip Accelerometry among U.S. Older Adults: How Many Days Are Enough?
Source: PLoS One. 2017 Mar 22;12(3):e0174739. doi: 10.1371/journal.pone.0174739 (PMC5362229; doi:10.1371/journal.pone.0174739)

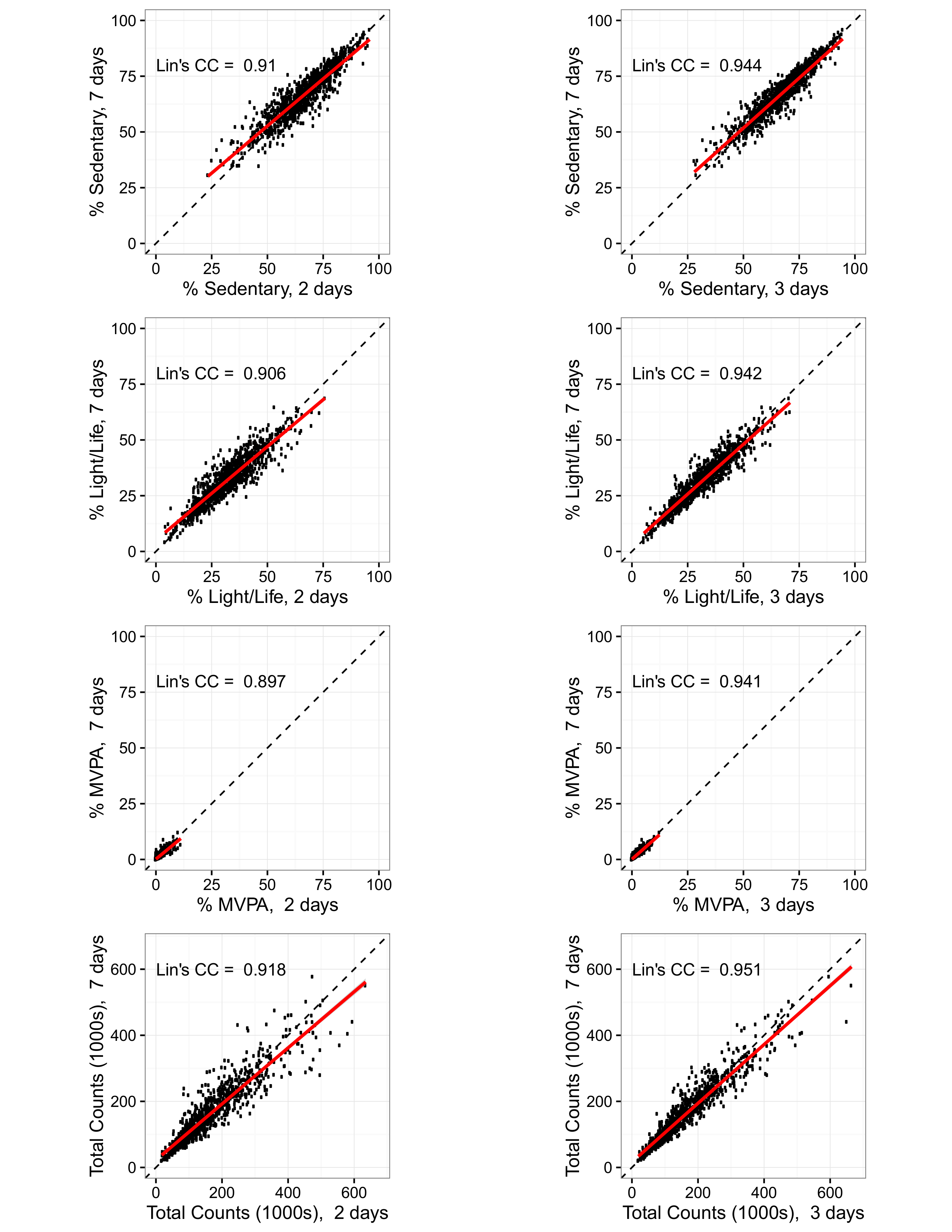

Supplement: S2 Fig — Lin's concordance correlation coefficients between 2-day (A) and 3-day (B) average percent of time spent in sedentary, light-lifestyle, and moderate-vigorous activity per daily versus 7-day estimate among adults aged 65 and older in National Health and Nutrition Examination Survey 2003±4 and 2005±6 accelerometry sub-study. (TIFF) [file pone.0174739.s001.tiff]
